# Case report: Plasma exchange as a therapy for Miller-Fisher syndrome

**DOI:** 10.3389/fimmu.2025.1460034

**Published:** 2025-02-18

**Authors:** Dongmei Guan, Yuanzhuang Shan, Hailin Zhang

**Affiliations:** ^1^ First Clinical Medical College, Shandong University of Traditional Chinese Medicine, Jinan, China; ^2^ Department of Neurology, Jining First People's Hospital, Jining, China; ^3^ Department of Neurology, Changsha Hospital of Traditional Chinese Medicine (Changsha Eighth Hospital), Changsha, China

**Keywords:** Miller-Fisher syndrome, ophthalmoplegia, Guillain-Barré syndrome, case report, plasma exchange

## Abstract

This is a report of an anti-GQ1b antibody-negative case of Miller-Fisher syndrome, presenting with diplopia and gait disturbance 3 days after catching a cold, accompanied by dizziness and headache, pain in the eyeballs and orbits, superficial sensory loss, ptosis, eye movements fixed in the middle of eyes, and limb weakness. Physical examination suggested that the patient had eye movements fixed in the middle of eyes, limb muscle strength was reduced, and the bilateral finger-to-nose and heel-knee-shin tests were clumsy and dysmetric. Assistant examinations showed albumin-cytologic dissociation in cerebrospinal fluid, incomplete bilateral facial nerve lesions, and negative anti-GQ1b antibody. After being treated with plasma exchange, the patient experienced slight improvement in eye adduction when she was discharged from the hospital and her diplopia and limb weakness were significantly improved. She could walk on her own without dizziness or headache and the pain in her eyeballs and orbits was completely relieved.

## Introduction

Miller-Fisher syndrome (MFS) is considered a rare variant of Guillain-Barré syndrome (GBS), characterized by the typical triad of areflexia or hyporeflexia, ataxia, and ophthalmoplegia, or accompanied by other symptoms, such as facial paralysis, limb weakness, optic neuritis, hypoesthesia, etc. MFS with eye movements fixed as a clinical manifestation is relatively rare in clinical practice. I hereby report a case of event-free survival with eye movements fixed, diplopia, gait disturbance, pain in the forehead and eyes and negative results from nerve conduction study, cerebrospinal fluid (CSF) analysis and anti-GQ1b antibody test to enhance clinicians’ understanding of the disease by reviewing and analyzing the patient’s clinical data, diagnosis and treatment process, efficacy and adverse reactions.

## Case report

The patient, a 53-year-old female went to the emergency room of Jining No.1 People’s Hospital on November 23, 2023, complaining of “diplopia and gait disturbance for 3 days.” The patient had diplopia and gait disturbance after catching a cold 3 days ago accompanied by dizziness and headache, and limb weakness, which was obvious when moving, but without motor dysfunction. She had tests in the local hospital including electrocardiogram, glycosylated hemoglobin, thyroid function, procalcitonin, five items of coagulation, five items of hepatitis B, liver function, kidney function, bilirubin, antistreptolysin O, complement, blood lipids, myocardial enzyme spectrum, uric acid, electrolytes, blood sugar, homocysteine, and rheumatoid factor: all were basically normal. Blood routine showed a neutrophil count of 8.96×10^9/L, neutrophil percentage of 85.3% and CRP l level of 8.9mg/L (**Table 1**). No obvious abnormalities were found in the brain MRI scan, which was consistent with the MRA manifestations of cerebral arteriosclerosis. As the treatment was unsatisfactory, she came to our hospital with symptoms upon admission: diplopia, gait disturbance, accompanied by dizziness and headache, limb weakness, pain in the eyeballs and orbits, superficial sensory loss, bilaterial ptosis, without muscle weakness related to the fluctuation phenomenon of “light in the morning and heavy in the evening”, without dysphagia or motor dysfunction or numbness, etc. She had undergone a parotidectomy 4 months ago, with an unremarkable personal and family history.

**Table 1:** Laboratory data visualization.

Complete blood count:neutrophils 8.96×10^9/L Neutrophil percentage 85.3%	CRP:8.9mg/L	Complete blood count:leukocytes 10.04×10^9/L lymphocytes 3.51×10^9/L
Routine examination of cerebrospinal fluid:Pressure 120 mmH_2_0 colorless clear and transparent leukocyte count 4×10^6/L WBC occasionally seen	Immunoglobulins: IgAC 8.12mg/L IgGC 66.00mg/L IgMC 1.98mg/L protein assay 0.60g/L	Nerve conduction studies (NCS):Incomplete damage to the lateral nerves bilaterally and no significant damage to the nerves of the extremities
Ink staining, fungal smear, antacid bacillus test, TORCH-IgM/IgG, and novel cryptococcal antigen assay showed no significant abnormalities	Ganglioside XII antibody test was negative	

**Figure 1:**
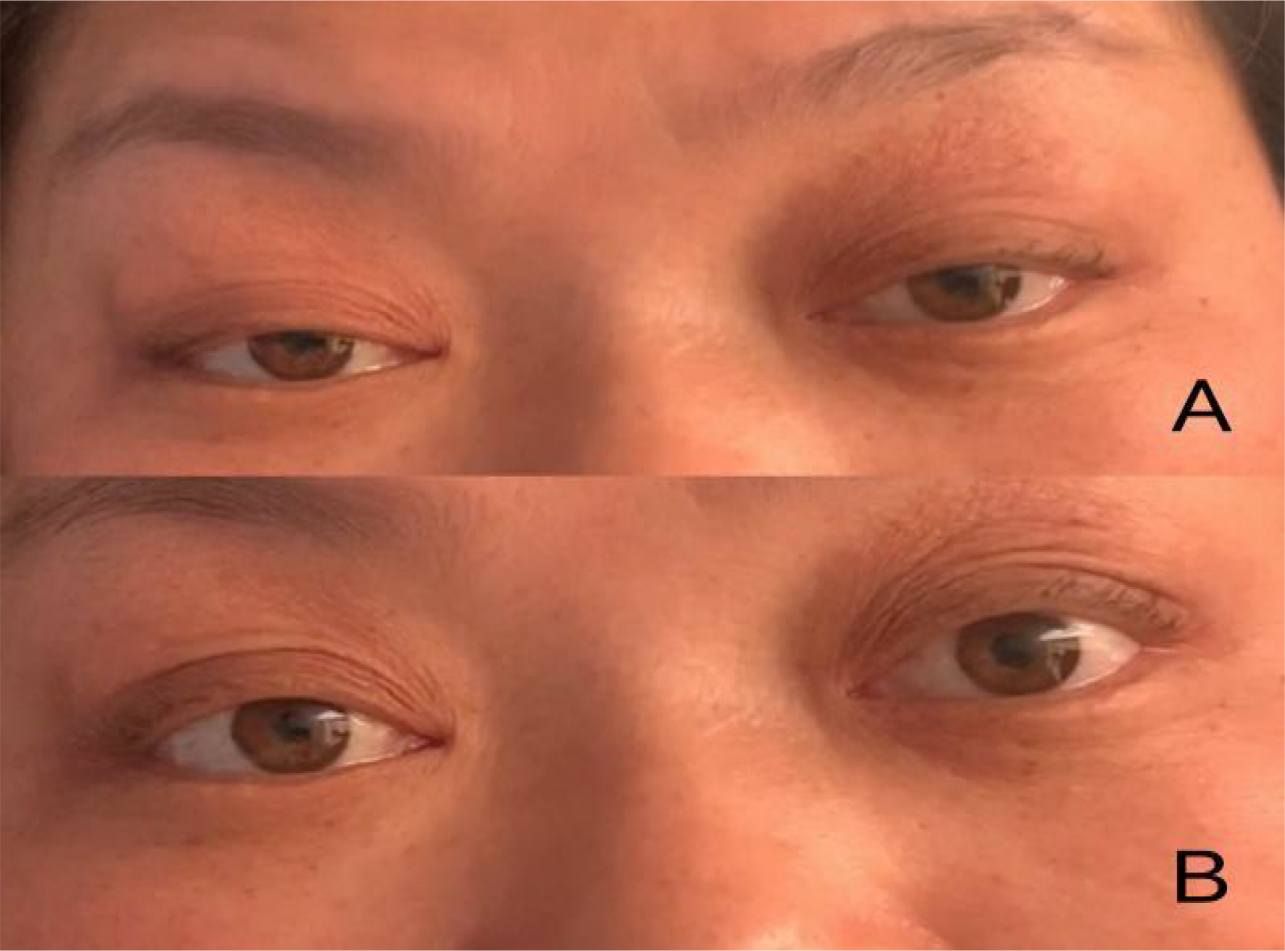
**(A)** Bilateral ptosis and eye movements fixed in the middle of eyes upon admission; **(B)** After conducting PE for 2 times.

Physical examination upon admission: T 36.6°C, P 68/min reg, R 22/min and BP 132/84mmHg. No obvious abnormalities were found in the heart, lungs and abdomen. The patient was conscious and fluent in speech. The gross measurement of advanced IQ test was normal. The bilateral pupils were equal in size and round, sensitive to light reflex, and eye movements were fixed in the middle of eyes (see **Figure 1**). She had normal facial sensation, symmetrical forehead lines and nasolabial folds, normal pharyngeal reflex, grade 5 muscle strength in the limbs and normal muscle tone in the limbs. The bilateral finger-to-nose and heel-knee-shin tests were clumsy and dysmetric, but the sensation in the limbs and trunk was normal, deep tendon reflexes were equal bilaterally in the upper and lower extremities (+), the bilateral Babinski signs were negative and no meningeal irritation signs were elicited.

**Figure 2:**
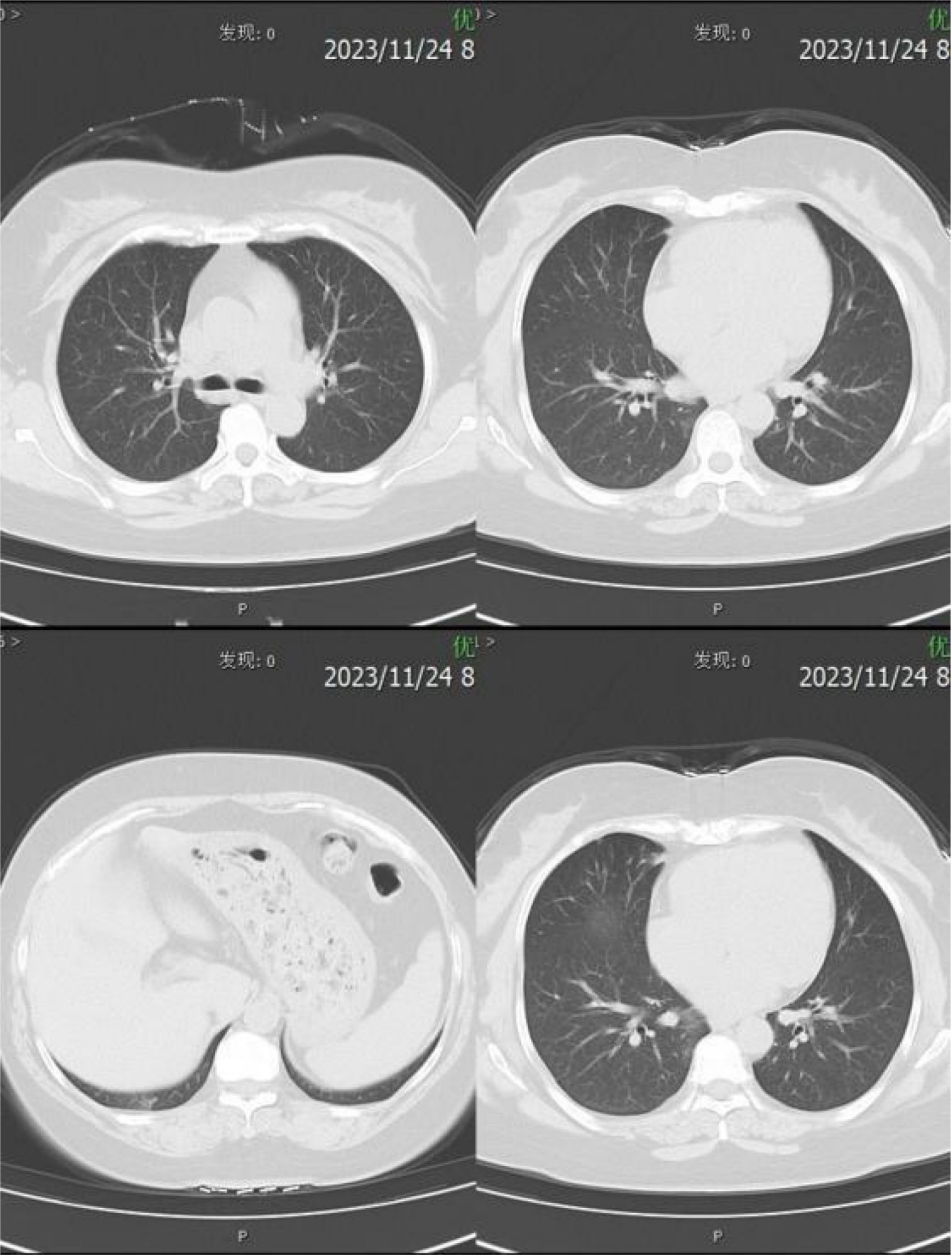
CT imaging of the chest.

**Figure 3:**
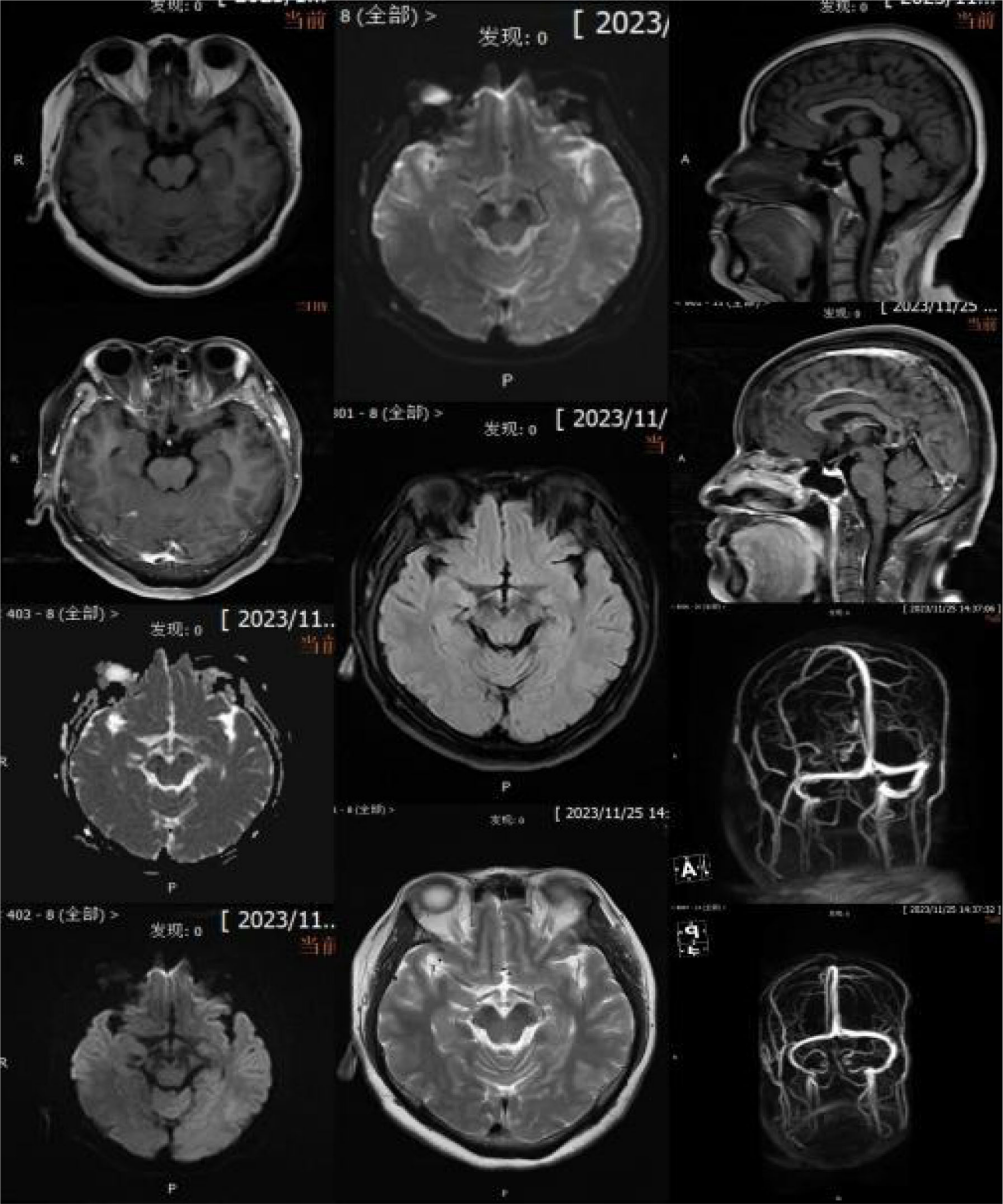
Brain MRI scan + contrast-enhanced + MRV.

Relevant assistant examinations upon admission: no obvious abnormalities in hepatitis C, syphilis, HIV, liver function, myocardial enzyme spectrum, electrolytes, kidney function, and five items of coagulation; blood routine showed a white blood cell (WBC) count of 10.04×10^9/L and lymphocytes count of 3.51×10^ 9/L. Lumbar puncture demonstrated a CSF (colorless and clear) pressure of 120 mmH20, WBC count of 4×10^6/L and WBC may present occasionally in urinalysis; immunoglobulins: IgAC 8.12mg/L, IgGGC 66.00mg/L, IgMC 1.98mg/L and protein concentration 0.60g/L; and no obvious abnormalities in ink staining, fungal smear, acid-fast bacilli test, TORCH-IgM/IgG, and detection of Cryptococcus neoformans antigens. Nerve conduction study (NCS) showed incomplete bilateral facial palsy and no obvious peripheral neuropathy. Twelve antibodies of peripheral serum gangliosides tested negative. Chest CT scan showed slight inflammation in lungs, a few nodules in lungs, calcification in the left lung, focal pleural thickening, and multiple calcifications in the liver (see **Figure 2**). Brain MRI scan + contrast-enhanced + MRV suggested a few ischemic degeneration lesions in the brain and slight inflammation in paranasal sinuses, but no orbital space-occupying lesions, cavernous fistula, or cerebral venous sinus thrombosis (CVST) were found (see **Figure 3**).

**Figure 4:**
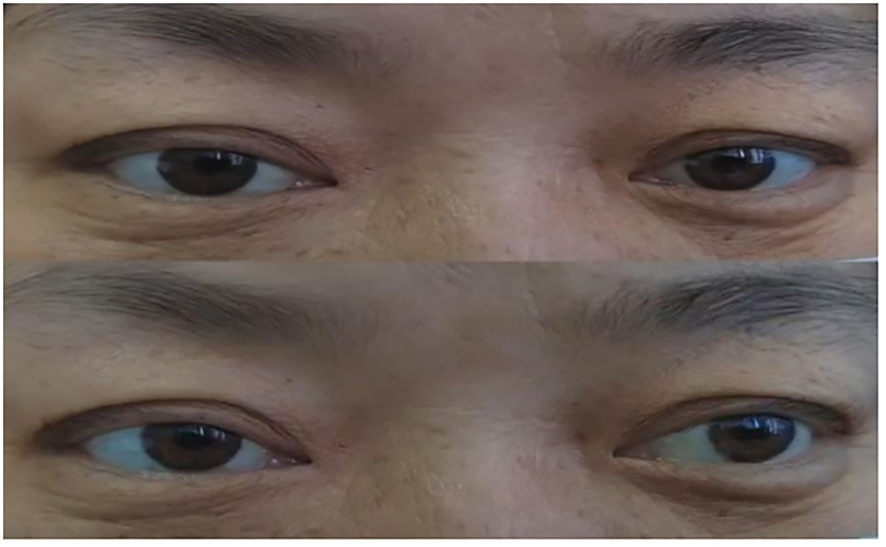
Two weeks after discharge. (Eyes were able to abduct, but unable to adduct fully).

**Figure 5:**
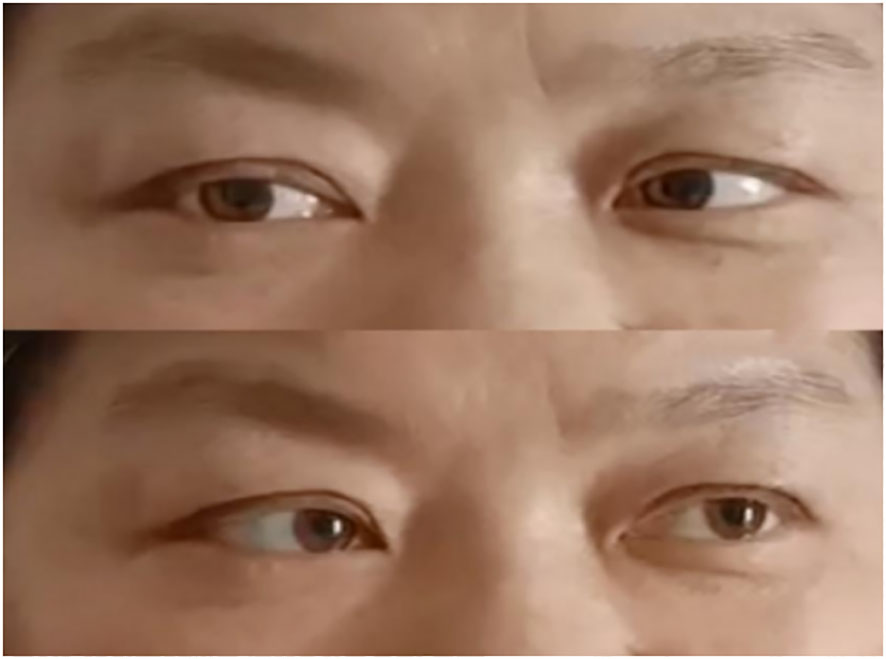
After 1.5 months of follow-up. (Eyes were not able to abduct fully but can move in other directions).

**Figure 6:**
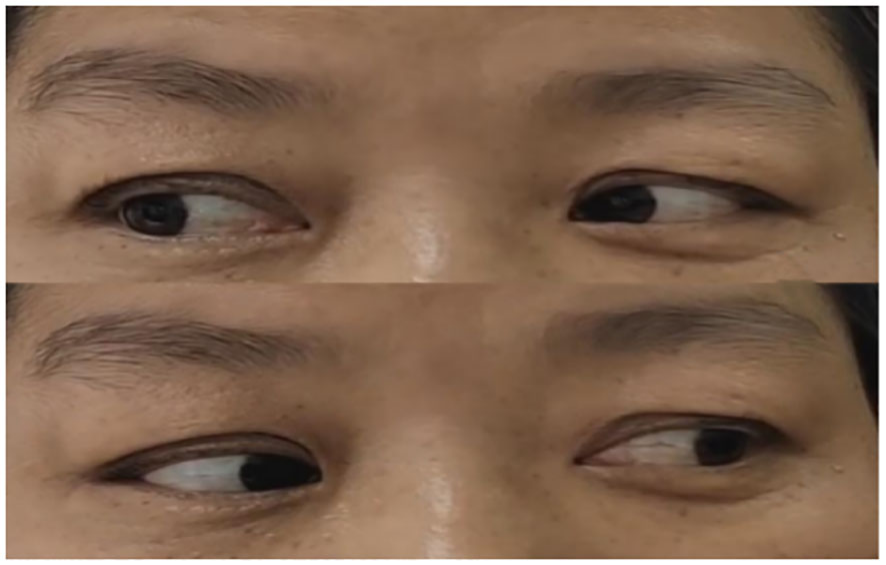
After 3.5 months of follow-up. (Eye movement returned to normal).

Combined with the patient’s medical history, symptoms, signs, and assistant examinations, she was diagnosed as MFS and treated with plasma exchange (PE), with details as follows: the patient weighed 76kg was performed with PE 2 days/time, 5 times in a row with the amount of each: 1950ml, 1921ml, 2270ml, 2370ml and 2420ml; at the same time, symptomatic treatment such as nerve nutrition and analgesia were provided. During the 1st and 2nd PE, rashes and itching appeared on her hands, which were relieved after taking cetirizine. After completing the 5th PE, the patient’s eye movements were still fixed in the middle of eyes, and her diplopia, ptosis and limb weakness improved significantly. She could walk on her own without dizziness or headache and the pain in her eyeballs and orbits was completely relieved. After 15 days, she was discharged from the hospital after the symptoms improved. Neurological examination showed that her eyes were still unable to abduct, with slight improvement in eye adduction; deep tendon reflexes were equal bilaterally in the upper and lower extremities (+) and the bilateral finger-to-nose and heel-knee-shin tests were clumsy and dysmetric. An outpatient reexamination 2 weeks after discharge revealed that the patient still had diplopia and epiphora, and her eyes were able to abduct, but unable to adduct fully (see **Figure 4**). After 1.5 months of follow-up, she still had diplopia which was less severe than before. Her eyes were unable to abduct fully without other positive signs (see **Figure 5**). After 3.5 months of follow-up, her diplopia disappeared, and no obvious positive signs were found in the neurological examination (see **Figure 6**).

## Discussion

GBS is an immune-mediated, degenerative demyelinating neurological disorder, characterized by progressive, symmetrical ascending paralysis with sensory impairment and dysautonomia. MFS is one of the rare subtypes of GBS, with a worldwide prevalence of one in 100,000. Asians have higher rates of MFS than Western populations. It accounts for approximately 1% to 7% of GBS in the Western population, while it ranges from approximately 19% to 25% in the Asian population (1). It has similar pathogenesis as GBS with most patients reported an infectious illness prior to nervous system involvement, and about 1/3 of patients have no history of antecedent infection (2). Major clinical manifestations include eyestrain symptoms, ataxia and areflexia. Eye symptoms previously reported included external ophthalmoplegia, diplopia, pupil dilation, etc. characterized by eye movements fixed and ptosis. In 2014, experts proposed new diagnostic criteria for MFS, including core clinical features (ataxia, ophthalmoplegia, areflexia or hyporeflexia) and supporting features (history of infection, CSF and anti-GQ1b antibody tests, etc.). For patients without the triad, they are diagnosed as incomplete MFS if anti-GQ1b antibodies are positive (3). The patient herein met the typical triad, and the supporting features included a history of infection and albumin-cytologic dissociation in CSF. Although the serologic test showed anti-GQ1b antibodies were negative, the antibody positivity can only be used as supporting evidence but as an exclusion criterion or basis for the diagnosis. The patient was diagnosed with MFS based on the clinical and supporting features, combined with the theory that the diagnosis of MFS relies more on clinical symptoms. In addition, the treatment is so effective because of the support and cooperation of nursing (See **Table 2**).

**Table 2:** Timeline with relevant data from the episode of care.

2023.11.23	T:36.6°C P:68 beats/min R:22 beats/min BP:132/84mmHg Bilateral eye fixation, centered in the orthostatic position, bilateral finger-nose and heel-knee-tibia tests were unstable and accurate, the rest was not special.
2023.11.25	Initial diagnosis of MFS
2023.11.27	Central venous cannulation, cardiac monitoring, oxygen saturation monitoring, first PE started at 15:10, finished at 16:29, replacement 1950ml
2023.11.29	Cardiac monitoring, oxygen saturation monitoring, second PE started at 09:12, finished at 10:41, replacement of 1921 ml
2023.12.01	Cardiac monitoring, oxygen saturation monitoring, third PE started at 09:08, finished at 10:30, 2270 ml replaced
2023.12.03	Cardiac monitoring, oxygen saturation monitoring, fourth PE started at 12:35, finished at 14:00, 2370 ml replaced
2023.12.05	Cardiac monitoring, oxygen saturation monitoring, fourth PE started at 10:43 and ended at 12:15 with 2420 ml of replacement
2023.12.07	Ocular abduction was not possible, adduction was slightly better than before, upward and downward vision was not possible, tendon reflexes of the limbs were bilaterally isokinetic (+), and bilateral finger-nose and heel-knee-tibia tests were stable and accurate.
2023.12.21	Follow-up 2 weeks after discharge, review of vision, occasional tearing, both eyes may be abducted and insufficiently adducted, the rest is unexceptional.
2024.01.22	On follow-up 1.5 months after discharge, the patient still had retests to a lesser extent than before, with insufficient abduction in both eyes and no other positive signs remaining.
2024.03.21	On follow-up 3.5 months after discharge, the patient’s diplopia disappeared, and neurologic examination showed no significant positive signs.

Among the various subtypes of GBS spectrum, each subtype has autoantibodies against different glycolipids of gangliosides, such as GQ1b, GM1, GM2, GD1a/b, etc. Anti-GQ1b antibodies are common biological markers of MFS. Serum anti-GQ1b antibodies have high sensitivity and specificity. More than 88% of patients with MFS are tested positive for anti-GQ1b antibodies, while CSF anti-GQ1b antibody has a sensitivity of 20% and a specificity of 100%. GQ1b is a ganglioside present in the paranodal myelin while anti-GQ1b antibodies directly affect the neuro-ocular junction. The accumulation of anti-GQ1b antibody in cranial nerves (III, IV and VI) is the major cause of ophthalmoplegia (4). The anti-GQ1b antibodies of the patient herein were tested negative. It is possible that pathogenic antibodies were small in volume but highly viral, causing severe neuropathic damage, resulting in typical clinical manifestations but negative antibody tests. Zhang et al. reported a case of MFS, which started with dizziness and diplopia and showed external ophthalmoplegia, absence of tendon reflexes, and ataxia, no albumin-cytologic dissociation in CSF, negative anti-GQ1b antibodies after having physical examination combined with clinical symptoms (5). A pathological study found that patients with MFS had patchy and segmental demyelination related to the invasion of foamy macrophages and lymphocytes in the peripheral nerves and cranial nerves (6). It has been reported that among all the patients who underwent lumbar puncture, only 37% of them underwent in the first week of disease onset had albumin-cytologic dissociation, the percentage improved to 76% in the second week (7). Therefore, MFS still cannot be ruled out, even though testing results showed negative for albumin-cytologic dissociation. Results of lumbar puncture 6 days after onset showed: albumin-cytologic dissociation, IgAC 8.12mg/L, IgGGC 66.00mg/L, IgMC 1.98mg/L and elevated protein 0.60g/L, providing basis for diagnosis.

MFS is not generally unassociated with abnormalities in brain imaging. A study reported that hyperintensity in T2WI at the level of the brainstem and the cranial and spinal nerve roots can be found in 2% of cases, providing evidence supporting central involvement (8). The brain MRI scan + contrast-enhanced + MRV of the patient herein showed a few ischemic degeneration lesions in the brain, which can be used to differentiate it from other diseases, such as acute cerebrovascular disease, brain tumor, formation of CVST, multiple sclerosis, and neurosarcoidosis. The disease is also required to be differentiated from diseases including ocular myasthenia gravis and pharyngeal-cervical-brachial variant. The case should be distinguished from Bickerstaff brainstem encephalitis (BBE), a rare variant of MFS with clinical manifestations including signs of ophthalmoplegia, ataxia, disorder of consciousness and/or pyramidal tract damage, while MFS shows no symptoms of disorder of consciousness or pyramidal tract damage. As the NCS of the patient herein indicated incomplete bilateral facial nerve lesions without any signs of decrease in sensory nerve action potential or absence of H-reflex in four limbs, GBS can be ruled out. NCS abnormalities were uncommon in MFS.

Controversy still exists in the treatment of MFS, a self-limited disease. Studies have shown that treatment does not shorten recovery time. Depending on the severity and other comorbid factors, symptoms may resolve on their own, with an average resolution time of 1-6 months (4). Immunotherapy includes PE and intravenous immunoglobulin (IVIG). IVIG neutralizes pathogenic antibodies by regulating the activation and effector functions of B and T cells. Some studies reported that IVIG can relieve symptoms and make recovery earlier but cannot shorten recovery time. PE works by clearing pathogenic antibodies from patients’ blood but is rarely applied in clinical practice. After conducting PE for 5 times, the patient herein experienced slight improvement in eye adduction, but was unable to abduct. Deep tendon reflexes were equal bilaterally in the upper and lower extremities (+) without other symptoms. A study reported a pregnant woman presenting at 27 weeks of gestation with MFS, treated with PE with a satisfactory course after 15 days, continuation of normal pregnancy and delivery of a healthy neonate (9). It was inconsistent with the statement that PE may be ineffective (10).

In conclusion, most patients with MFS have a favorable prognosis and a low recurrence. However, the disease has an acute onset and rapid progression, and a few patients may experience dyspnea or even death due to respiratory muscle involvement. Therefore, immunotherapy is the first line of defense, and PE or IVIG treatment is advised as early as possible according to patients’ financial conditions. By removing abnormal autoantibodies and inflammatory factors from the plasma, PE reduces the inflammatory response of the nerves, reduces the progression of the disease, promotes neurological recovery, shortens the recovery time, and improves the quality of life. However, there are some limitations and a comprehensive assessment is needed based on the individual patient’s condition.

## Data Availability

The datasets presented in this article are not readily available because of ethical and privacy restrictions. Requests to access the datasets should be directed to the corresponding author.
